# *Caenorhabditis elegans* NHR-14/HNF4α regulates DNA damage-induced apoptosis through cooperating with *cep-1*/p53

**DOI:** 10.1186/s12964-022-00920-5

**Published:** 2022-09-01

**Authors:** Lei Sang, Rui Dong, Rui Liu, Qinggang Hao, Weiyu Bai, Jianwei Sun

**Affiliations:** 1 Center for Life Sciences, School of Life Sciences, State Key Laboratory for Conservation and Utilization of Bio-Resources in Yunnan, Yunnan University, Kunming, China; 2 The Third Affiliated Hospital of Kunming Medical University, Kunming, China

**Keywords:** NHR-14, CEP-1/p53, DNA damage, Apoptosis, *Caenorhabditis elegans*

## Abstract

**Background:**

Nuclear hormone receptors are involved in transcriptional regulation and many important cellular processes including development and metabolism. However, its role in DNA damage-induced apoptosis remains elusive.

**Methods:**

Synchronized young adult animals were irradiated with different doses of gamma-Ray, and then put back to culture at 20 °C. Germline cell apoptosis was scored at different time point.

**Results:**

Deletion of *nhr-14* led to decreased DNA damage-induced germline apoptosis, but not the physiological programmed cell death. We also demonstrate that *nhr-14* functions downstream of the DNA damage checkpoint pathway. Moreover, we show that *nhr-14* regulates *egl-1* and *ced-13* transcription upon DNA damage. Mechanistically, NHR-14 forms a complex with CEP-1/p53 and binds directly to the *egl-1* promoter to promote egl-1 transcription..

**Conclusions:**

Our results indicate that NHR-14/HNF4α cooperates with CEP-1/p53 to regulate DNA damage-induced apoptosis.

**Graphic abstract:**

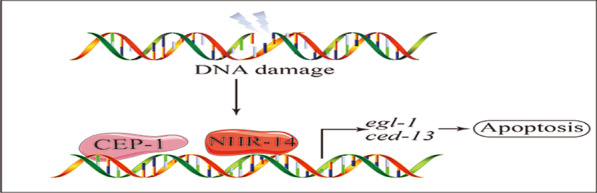

**Video abstract**

**Supplementary Information:**

The online version contains supplementary material available at 10.1186/s12964-022-00920-5.

## Background

Nuclear hormone receptors (NHRs) comprise a large family of transcription factors distinguished by a highly conserved DNA binding domain and a structurally conserved ligand-binding domain. There are 284 predicted NHR genes in *C.elegans* [1]*.* Nuclear hormone receptors have been shown to regulate important developmental process [2–5]. The nuclear hormone receptor NHR-6 is required for spermatheca development [6, 7]. NHR-86 controls anti-pathogen responses [8], and NHR-49 controls fat consumption and fatty acid composition in *C. elegans* [9]. NHR-14, an orphan receptor, has been reported to regulate innate immunity and iron uptake [10]. However, the role of NHR-14 in programmed cell death has not been documented.

Programmed cell death (i.e., apoptosis) is one of the most important processes in the metazoans development. It plays key roles in animal development and DNA damage repair. DNA damage-induced apoptosis is cell death after severe DNA damage, which is associated with a number of human diseases including cancer. *Caenorhabditis elegans* has been used extensively to study programmed cell death induced by DNA damage responses. We previously demonstrated that *prmt-5*, the *C. elegans* homolog of mammalian type II protein arginine methyltransferase PRMT5, negatively regulates DNA damage-induced apoptosis [11]. *prmt-5(gk357)* deletion mutants have increased germline programmed cell death after DNA damage. Furthermore, genetic analyses indicated that *prmt-5*-mediated apoptosis depends on *cep-1*/p53 and requires the core cell death pathway. In *C. elegans*, the p53 homolog CEP-1 acts as a key effector to mediate germ cell apoptosis triggered by ionizing irradiation [12]. Although many factors have been reported to be involved in p53/*cep-1*-dependent apoptotic pathway, the details of this pathway are yet to be completely understood.

In the present study, we show that RNAi knockdown of *nhr-14* suppresses DNA damage-induced apoptosis in *prmt-5(gk357)* deletion mutants. Further, we show that *nhr-14* is a new factor involved in DNA damage-induced apoptosis and that *nhr-14* is not a checkpoint gene and functions downstream of the checkpoint genes. Our study confirmed that NHR-14 cooperates with CEP-1/p53 to regulate *egl-1* (Bcl-2 homology region 3 domain containing gene) and *ced-13* (Bcl-2 homology region 3 domain containing gene) expression and DNA damage-induced apoptosis, which reveals a novel role and mechanism for NHR-14/HNF4α in apoptosis. Dysregulation of DNA damage induced apoptosis has been reported to closely correlated tumorigenesis. Our study might provide new strategy and targets for prevention and therapy of tumor.

## Methods

### *C. elegans* strains and genetics

The strains of *nhr-14(tm1473)*, *brc-1(tm1145)*, *vps-18(tm1125)* were provided by Dr. Shohei Mitani. *prmt-5(gk357)*, *cep-1(gk138)*, *gld-1(op236)*, *akt-1(ok525)*, *abl-1(ok171)*, *ced-9(n1653)*, *hus-1(op244)* and *clk-2(mn159)* strains were provided by *C. elegans* Genetic Center (CGC). which is funded by NIH Office of Research Infrastructure Programs (P40 OD010440). FU112: *prmt-5(gk357)*; *nhr-14(tm1473)*, FU144: *ced-1(e1375)*; *nhr-14(tm1473)*, FU41: *ced-1(e1375)*; *prmt-5(gk357)*, FU173: *ced-1(e1375)*; *prmt-5(gk357)*; *nhr-14(tm1473)*, FU312: *akt-1(ok525)*; *nhr-14(tm1473)*, FU509: *ced-9(n1653)*; *nhr-14(tm1473)*, FU279: *brc-1(tm1145)*; *nhr-14(tm1473)*, FU718: *hus-1(op244)*; *nhr-14(tm1473)*, FU720: *clk-2(mn159)*; *nhr-14(tm1473)* and FU150: *gld-1(op236)*; *nhr-14(tm1473)* were provided by Dr. Chonglin Yang. Worms were cultured and maintained using standard procedures. The Bristol N2 strain was used as wild type. Deletion strains were outcrossed with N2 strains for 6 times. Double mutants were constructed with standard protocols.

### Germ cell apoptosis assay

Synchronized young adult animals were irradiated with gamma-Ray (120 Gy), which was located in the Peking University Health Science Center. Irradiated animals were put back to culture at 20 °C at different time points. Worms with normal germline morphology were scored for germline cell apoptosis with a DIC Zeiss microscope. The apoptotic cells showed button-like morphology under the DIC microscope and the number of apoptotic cells were scored.

### Radiation sensitivity assay

N2 wild-type worms, *nhr-14(tm1473)*, *hus-1(op244)*, *hus-1(op244)*; *nhr-14(tm1473)* double mutant, *clk-2(mn159)*, *clk-2(mn159)*; *nhr-14(tm1473)* double mutant worms were irradiated respectively at the L4 stage as indicated. Eggs laid 8–24 h after irradiation (corresponding to pachytene-stage germ cells at the time of irradiation) were counted. Surviving offspring animals were counted for days 1 and 2. The result represents the percent of survival of embryos of six different animals per strain.

### Mammalian cell culture, transfection and immunoprecipitation

Human embryonic kidney (HEK293) cells were grown in Dulbecco’s modified Eagle’s medium (HyClone) supplemented with 10% fetal bovine serum (HyClone). The transfection was performed with 2.0 ug of mammalian vectors expressing worm proteins with different tags (i.e., pCMV-myc-*cep-1*, pCMV-tag2B-*nhr-14*) using PEI reagent. After 36 h of transfection, cells were harvested and lysed in a buffer containing 50 mM Tris (pH 8.0), 150 mM NaCl, 0.5% sodium deoxycholate, 1% Triton X-100, 1 mM phenylmethylsulfonyl fluoride (PMSF). The lysate was incubated with anti-Flag antibody (M2)-conjugated agarose beads (Sigma) for more than 2 h at 4 °C. The beads were washed extensively in a buffer containing 50 mM Tris (pH 8.0), 150 mM NaCl, 1 mM PMSF and 1% NP-40. Bound proteins were eluted and resolved on sodium dodecyl sulfate polyacrylamide gel electrophoresis (SDS-PAGE) and detected with Western blot assay.

### Western blot assay

Cells were scraped and lysed in lysis buffers on ice for 15 min, 15 μg total proteins were loaded on SDS-PAGE gels as co-immunoprecipitation experiment input. The SDS-PAGE gel was first run on 60 V for 30 min and then 120 V until the dye ran out of the gel, then the protein was transferred to PVDF membranes. The membranes were blocked in 5% non-fat dry milk in Tris-buffered saline, 0.05% Tween for 30 min at room temperature, and then incubated with primary antibodies for 2–4 h at 4 °C, followed by incubation with secondary antibodies for 60 min at room temperature. The results were detected by an ECL-plus Western blotting detection system (Tanon-5200Multi). The primary antibodies used in this study were as follows: anti-Flag (Sigma, Cat#:F3165); anti-Myc (Sigma, Cat#:HPA055893); GAPDH (Santa CruZ, Cat#: sc-32233).

### GST pull-down assay

For GST pull-down assay, purified GST or GST-CEP-1 fusion proteins were immobilized on glutathione-Sepharose beads and incubated with ^[35S]^methionine-labeled NHR-14 at 4 °C for more than 2 h. The beads were washed extensively and bound proteins were eluted and separated on 12% SDS-PAGE and exposed to phospho-imager (Amersham) for autoradiography.

### RT-qPCR assay

Total *C. elegans* RNA was extracted using TRIZOL methods and cDNA was synthesized using iScript cDNA Synthesis Kit (Bio-Rad Laboratories). qPCR was performed in an iCycler thermocycler (Bio-Rad Laboratories) using iQ SYBR Green Supermix (Bio-Rad Laboratories). mRNA levels were quantified using iCycler software (Bio-Rad Laboratories) and were normalized to *tbg-1*. The primers used for RT-qPCR were as follows:

- *egl-1* q-PCR NS: 5′-gattcttctcaatttgccgacg-3′;
- *egl-1* q-PCR CAS: 5′-tcatctgagcatcgaagtcatc-3′;
- *ced-13* q-PCR NS: 5′-acggtgtttgagttgcaagc-3′;
- *ced-13* q-PCR CAS: 5′-gtcgtacaagcgtgatggat-3′;
- *ced-3* q-PCR NS: 5′-ccaatttgttcagatgcatggg-3′
- *ced-3* q-PCR CAS: 5′-tctccgtgtgattcgtgtttg-3′
- *ced-4* q-PCR NS: 5′-acgcttatgatgtttttcaagtct-3′
- *ced-4* q-PCR CAS: 5′-cctcatctgacaaaacttcaacac-3′
- *ced-9* q-PCR NS: 5′-ctgtatcaggatgtggttcgg-3′
- *ced-9* q-PCR CAS: 5′-agcgatgtgtaaacgaagagg-3′
- *tbg-1*q-PCR NS: 5′-cgtcatcagcctggtagaaca-3′;
- *tbg-1*q-PCR CAS: 5′-tgatgactgtccacgttgga-3′.

All experiments were analyzed in triplicates.

### Genomic SELEX assay

SELEX assay was done according to our previous report [13]. *C. elegans* genomic DNA was digested by MseI, then MseI adaptor were ligated on both sides of the digested products. The DNA fragment library was amplified by PCR using MseI adaptor primers. After GST-NHR-14 (1–87 AA) and amplified DNA fragment library were incubated for 1 h, the nonspecific binding DNA fragments were washed off with washing solution, and then the specifically bound DNA fragments were amplified for the next round of SELEX screening. After 14 rounds of screening, the obtained protein specifically binds to the DNA binding domain of GST-NHR-14 DNA fragments were recovered, cloned into T vectors and sequenced. The obtained sequences were analyzed by meme software (http://meme.sdsc.edu/meme4_1/cgi-bin/meme.cgi).

### Statistical analysis

All the experiments were repeated three times and each experiment was performed in 3 replicates per sample. Data were analyzed using SPSS 19.0 and GraphPad Prism 6.0. Student’s t-test, Spearman correlation, Kaplan–Meier, log-rank test and Cox regression survival and Statistical significance was defined as *P < 0.05, **P < 0.01 or ***P < 0.001.

## Results

### Inactivation of *nhr-14*/HNF4α inhibits DNA damage-induced apoptosis

**Fig. 1:**
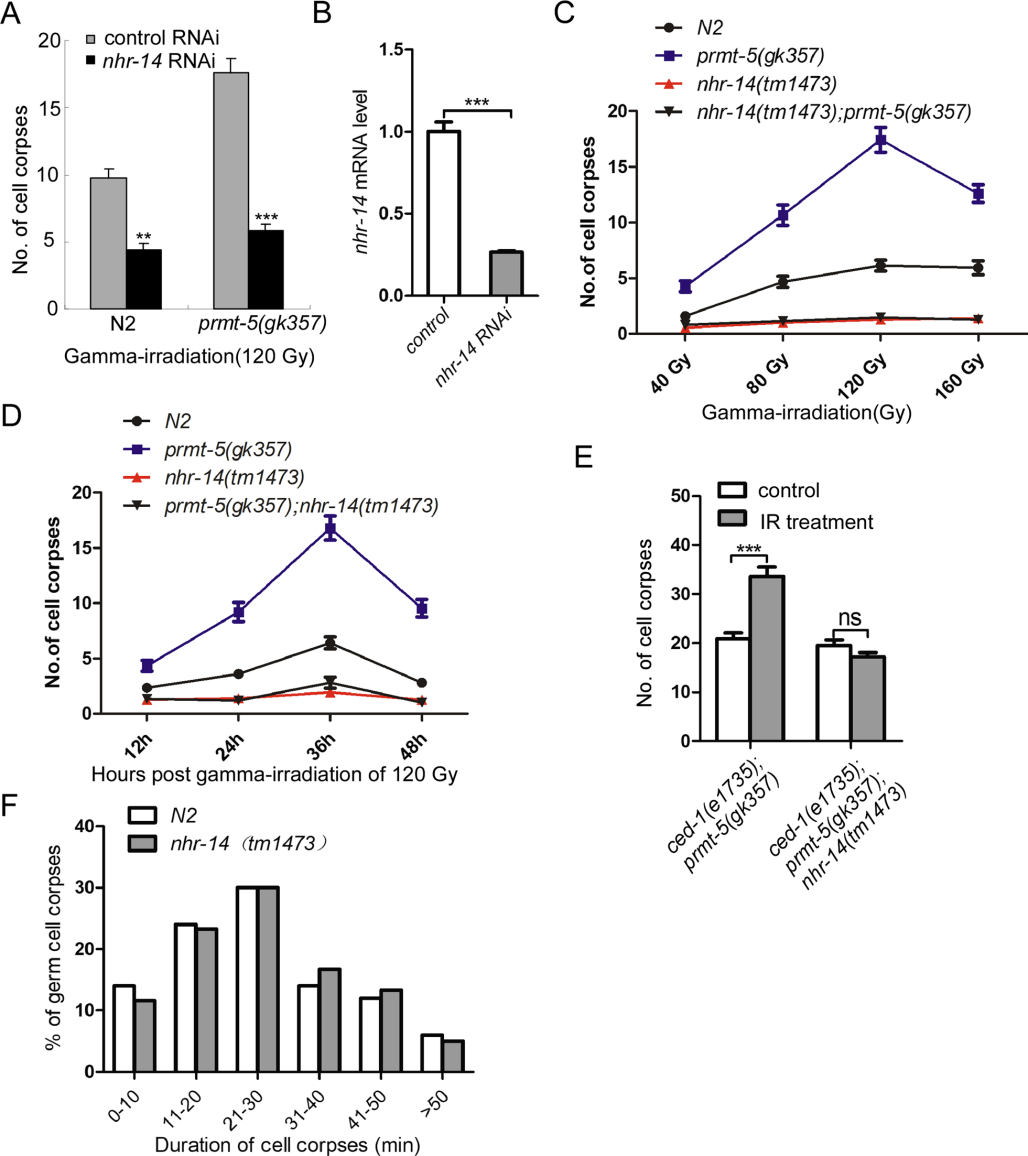
Inactivation of *nhr-14*/HNF4 inhibits DNA damage-induced programmed cell death in *prmt-5(gk357)*. **A** Quantitative analysis of germ cell apoptosis in control RNAi- and *nhr-14* RNAi-treated N2 and *prmt-5(gk357)* animals. N2 and *prmt-5(gk357)* were fed with control RNAi and *nhr-14* RNAi and then (L4) was irradiated. After 36 h of gamma-irradiation, germ cell apoptosis from one gonad arm of each animal were scored from at least 20 animals. Error bars represent standard error of the mean (SEM). ** and *** indicate p < 0.01 and 0.001, respectively. **B** q-PCR analysis of the nhr-14 RNAi efficiency. **C** Quantitative analysis of germ cell apoptosis induced by gamma-irradiation in N2, *nhr-14(tm1473)*, *prmt-5(gk357)* and *prmt-5(gk357)*; *nhr-14(tm1473)*. Germ cell apoptosis from one gonad arm of each animal were scored after 36 h of irradiation at indicated doses. At least 20 worms were scored at each radiation dose or time point. Error bars represent standard error of the mean (SEM). **D** Quantitative analysis germ cell apoptosis at indicated time points after irradiation (120 Gy) in N2, *nhr-14(tm1473)*, *prmt-5(gk357) and prmt-5(gk357)*; *nhr-14(tm1473)* animals. E. Quantitative analysis of germ cell apoptosis in *ced-1(e1375)*; *prmt-5(gk357)* and *ced-1(e1375)*; *prmt-5(gk357)*; *nhr-14(tm1473)* animals with and without IR treatment. *** indicate p < 0.001. F. Time lapse analysis of germ cell corpse duration in N2 and *nhr-14(tm1473)* upon DNA damage

To examine whether nuclear hormone receptor is directly involved in the regulation of DNA damage-induced apoptosis, we performed RNAi screen in the background of *prmt-5(gk357).* We found that knockdown of *nhr-14*/HNF4α reduced the DNA damage-induced programmed cell death in *prmt-5(gk357)* (Fig. 1A) after ionizing irradiation. *nhr-14* RNAi reduced about 75% of the *nhr-14* mRNA level (Fig. 1B). Further analysis showed that the *C. elegans nhr-14* gene is defined by the open reading frame T01B10.4 located on the linkage group X, and encodes a protein of 435 amino acids. The *nhr-14(tm1473)* deletion mutant contains a deletion of 409 bp in the third exon and third intron of *nhr-14*, and this deletion will result in an early stop of NHR-14 translation [10].

In order to test the role of *nhr-14*/HNF4α in DNA damage-induced apoptosis, we used *nhr-14(tm1473*) deletion mutants to analyze the germ cell apoptosis after ionizing irradiation. We found that *nhr-14(tm1473)* inhibited DNA damage-induced apoptosis in *prmt-5(gk357)* at different gamma-irradiation doses (Fig. 1C) and different times (Fig. 1D). In order to rule out that the decreased of apoptosis caused by DNA damage in *nhr-14(tm1473)* is due to a defect in cell corpse clearance, we analyzed the germ cell apoptosis in *ced-1(e1375)*; *prmt-5(gk357)* and *ced-1(e1375)*; *prmt-5(gk357)*; *nhr-14(tm1473)*. And the results showed that *nhr-14(tm1473)* still significantly inhibited DNA damage-induced apoptosis in *prmt-5(gk357)* in the background of *ced-1(e1735)* (Fig. 1E) We also performed a time lapse experiment and the result indicated that the cell corpses in *nhr-14(tm1473)* persisted the same time as in N2 (Fig. 1F). Our results suggested that *nhr-14* functions downstream of *prmt-5* and regulates DNA damage-induced programmed cell death.

**Fig. 2:**
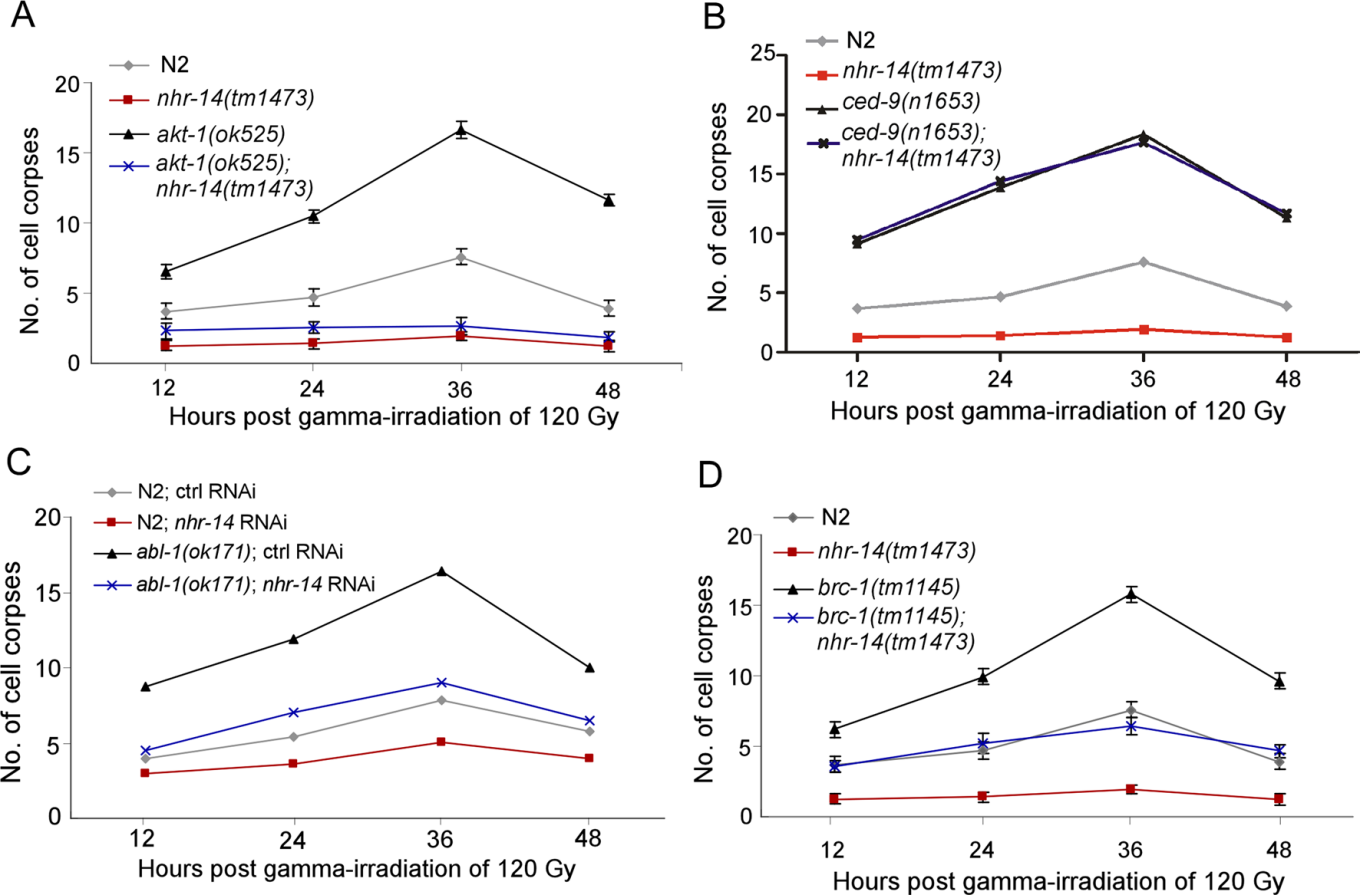
Epistasis analysis indicates that *nhr-14*/HNF4 mediated DNA damage-induced apoptosis. **A** Quantitative analysis of germ cell apoptosis induced by gamma-irradiation in N2, *nhr-14(tm147*3), *akt-1(ok525)* and *akt-1(ok525)*; *nhr-14(tm1473)*. Young adult animals were irradiated with gamma-ray (120 Gy) and analyzed at indicated time points after irradiation. Error bars represent standard error of the mean (SEM). **B** Quantitative analysis of germ cell apoptosis induced by gamma-irradiation in N2, nhr-14(tm1473), brc-1(tm1145) and brc-1(tm1145); nhr-14(tm1473) animals. D Quantitative analysis of germ cell apoptosis induced by gamma-irradiation in N2, nhr-14(tm1473), ced-9(n1653) and ced-9(n1653); nhr-14(tm1473) animals. **C** Quantitative analysis of germ cell apoptosis in control RNAi and nhr-14 RNAi-treated N2 and abl-1(ok171) animals. N2 and abl-1(ok171) were fed with control RNAi and nhr-14 RNAi and then (L4) was irradiated at 120 Gy. After 36 h of gamma-irradiation, germ cell apoptosis from one gonad arm of each animal were scored from at least 20 animals. Error bars represent standard error of the mean (SEM). **D** Quantitative analysis of germ cell apoptosis induced by gamma-irradiation in N2, nhr-14(tm1473), brc-1(tm1145) and brc-1(tm1145); nhr-14(tm1473) animals

To further determine whether *nhr-14*/HNF4α is a new factor involved in the DNA damage-induced cell apoptosis, we performed epistasis analysis using several well-defined cell survival molecules including AKT-1/AKT, ABL-1/ABL and CED-9/BCL2. Previous studies have demonstrated that loss-of-function mutation of *C. elegans akt-1(ok525)* exhibits dramatically increased programmed cell death after gamma-irradiation [14] and that mutation of *abl-1*/abl1 induces more germline apoptosis than wild type. Furthermore, it has been shown that loss-of-function of *ced-*9, a BCL-2 homolog in *C. elegans* [15], activates CED-3 to induce programmed cell death [16, 17] and that the *ced-9(n1653)* mutant exhibits more apoptotic cells upon DNA damage treatment. Our epistasis analysis revealed that *nhr-14*/HNF4α deletion abrogated DNA damage-induced apoptosis in *akt-1(ok525)* (Fig. 2A), but not in the *ced-9(n1653)* background (Fig. 2B). Knockdown of *nhr-14*/HNF4α led to dramatically decreased germline apoptosis in *abl-1(ok171)* mutants (Fig. 2C).

In addition, *brc-1* is the BRCA1 homolog in *C. elegans* and functions in DNA double-strand break repair after gamma-irradiation [18, 19]. Mutation of *brc-1*/BRCA1 resulted in failing to repair the double-strand break and induced germ cell apoptosis. We also found that the *brc-1(tm1145)*; *nhr-14(tm1473)* double mutant dramatically decreased germ cell apoptosis compared to *brc-1(tm1145)* alone after DNA damage (Fig. 2D).

Taken together, these findings indicate that *nhr-14*/HNF4α regulate DNA damage-induced programmed cell death in *C. elegans*.

### *nhr-14*/HNF4α does not affect physiological programmed cell death

**Fig. 3:**
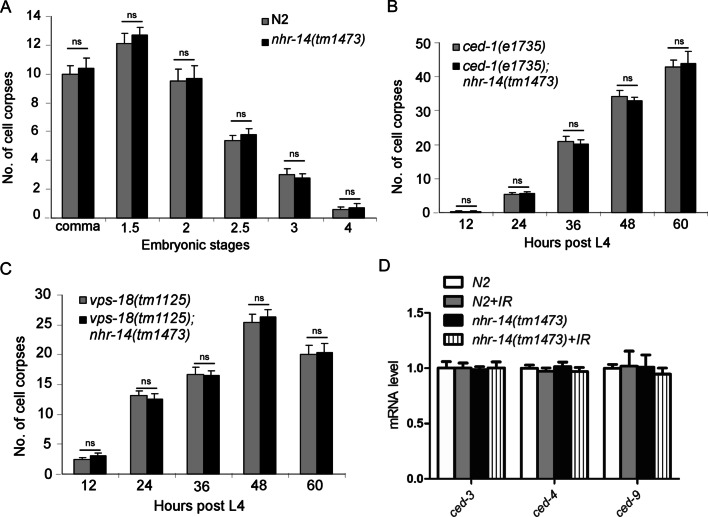
*nhr-14* does not affect the physiological programmed cell death. **A** Quantification of embryo cell apoptosis in N2 and *nhr-14(tm1473)* mutants. **B** Quantification of germline cell apoptosis in *ced-1(e1735)* and *ced-1(e1735)*; *nhr-14(tm1473)* animals at indicated time points post L4. **C** Quantification of germline cell apoptosis in *vps-18(tm1125)* and *vps-18(tm1125)*; *nhr-14(tm1473)* animals at indicated time points post L4. **D** q-PCR analysis *ced-3*, *ced-4* and *ced-9* mRNA levels in N2 and *nhr-14(tm1473)* at 36 h post L4

Since *nhr-14(tm1473)* showed less apoptosis upon gamma-irradiation, we next investigated the underlying cellular mechanism. We performed the time lapse phenotype analysis and found that there was no germline development defect and *nhr-14(tm1473)* showed the same apoptosis number as N2 at any time. These data indicate the decreased programmed cell death in *nhr-14(tm1473)* is neither due to germline development nor the delayed cell death. We further examined whether *nhr-14* affects the physiological programmed cell death in embryos. Figure 3A shows that there was no difference in the number of cell apoptosis in embryos between N2 and *nhr-14(tm1473)*. *ced-1*(*e1735*) [20] and *vps-18(tm1125)* [21] has been reported to affect cell corpse clearance. We also found no difference in the number of cell apoptosis in germline between wild type and *nhr-14(tm1473)* mutants in the background of *ced-1*(*e1735*) and *vps-18(tm1125)* (Fig. 3B, C). In order to further prove that *nhr-14* does not affect germline physiological programmed cell death, we analyzed the expression difference of *ced-3*, *ced-4* and *ced-9* in N2 and *nhr-14(tm1473)* by q-PCR, our results showed that *nhr-14* did not affect the mRNA levels of these three genes (Fig. 3D). These results indicate that *nhr-14*/HNF4α only affects the DNA damage-induced apoptosis, but not the physiological programmed cell death.

### *nhr-14*/HNF4α functions downstream of the checkpoint pathway

**Fig. 4:**
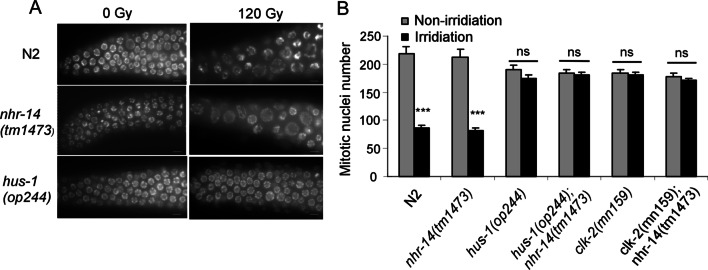
*nhr-14* is not a checkpoint gene and does not affect the cell cycle arrest after DNA damage. **A** Representative images of DAPI staining of the germline mitotic region in N2, *nhr-14(tm1473)* and *hus-1(op244)* worms. Young adult worms were treated with gamma-irradiation at 120 Gy. After 36 h of irradiation, germline was dissected and stained with DAPI. Bars, 5 μm. **B** Quantification of germline mitotic nuclear numbers after gamma irradiation in N2, *nhr-14(tm1473)*, *hus-1(op244)*, *hus-1(op234)*; *nhr-14(tm1473)*, *clk-2(mn159)*, *clk-2(mn159)*; *nhr-14 (tm1473)* worms. The gray and black bars represent nuclei numbers in the mitotic region in control and gamma irradiation-treated worm germline, respectively. *** indicate p < 0.001

Previous studies demonstrated that the checkpoint signaling pathways are activated upon DNA damage and play the critical role in repairing the damaged DNA or inducing programmed cell death [22, 23]. Mutations in checkpoint genes can restrain both DNA damage-induced cell cycle arrest and apoptosis upon gamma-irradiation in *C. elegans* [22]. Checkpoint mutants also showed embryonic lethality following gamma-irradiation [22]. HUS-1 is a *Caenorhabditis elegans* DNA damage checkpoint protein required for genome stability and CEP-1/p53-dependent activation of a BH3 domain protein in *C. elegans* [23].To determine where *nhr-14*/HNF4α functions in response to DNA damage, we first assessed the sensitivity of *nhr-14(tm1473)* mutants to gamma-irradiation using the radiation sensitivity assay. We found that the survival rate of *nhr-14(tm1473)* progeny was comparable to that of wild-type animals, but was much higher than that of checkpoint gene mutants *hus-1(op244)* and *clk-2(mn159)* (Table 1). In addition, *nhr-14(tm1473)* worms displayed similar cell cycle arrest in germline mitotic region to that in wild type following irradiation treatment (Fig. 4A). We further made *hus-1(op244)*; *nhr-14(tm1473)* and *clk-2(mn159)*; *nhr-14(tm1473)* double mutants, and found that these double mutants exhibited the same phenotype as the check point mutants (Fig. 4B). These results indicate that *nhr-14* is necessary for irradiation-induced apoptosis, but not for irradiation-induced cell cycle arrest. Our findings suggest that *nhr-14*/HNF4α is not involved in DNA repair and acts downstream of the checkpoint genes.

**Table 1:** *nhr-14* does not affect the survival of progeny after gamma-irradiation treatment

Irradiation	Survival (%)					
Dose (Gy)	*N2*	*nhr-14(tm1473)*	*hus-1(op244)*	*hus-1*; *nhr-14*	*clk-2(mn159)*	*clk-2*; *nhr-14*
0	100.0 ± 0	100.0 ± 0	97.6 ± 0.1	98.3 ± 0.8	98.0 ± 0.7	96.0 ± 1.5
40	86.3 ± 1.2	86.1 ± 1.8	37.7 ± 1.1	39.6 ± 2.0	29.1 ± 2.6	28.9 ± 1.7
80	76.7 ± 2.4	76.3 ± 1.8	18.7 ± 1.3	18.0 ± 1.2	9.4 ± 0.5	11.1 ± 0.8
120	64.5 ± 1.6	62.5 ± 1.4	3.2 ± 0.5	3.5 ± 0.7	2.8 ± 1.0	2.8 ± 1.0

The survival of *nhr-14(tm1473)* mutant progeny is not sensitive to irradiation

### NHR-14 cooperates with CEP-1/p53 to regulate *egl-1* and *ced-13* transcription upon DNA damage

**Fig. 5:**
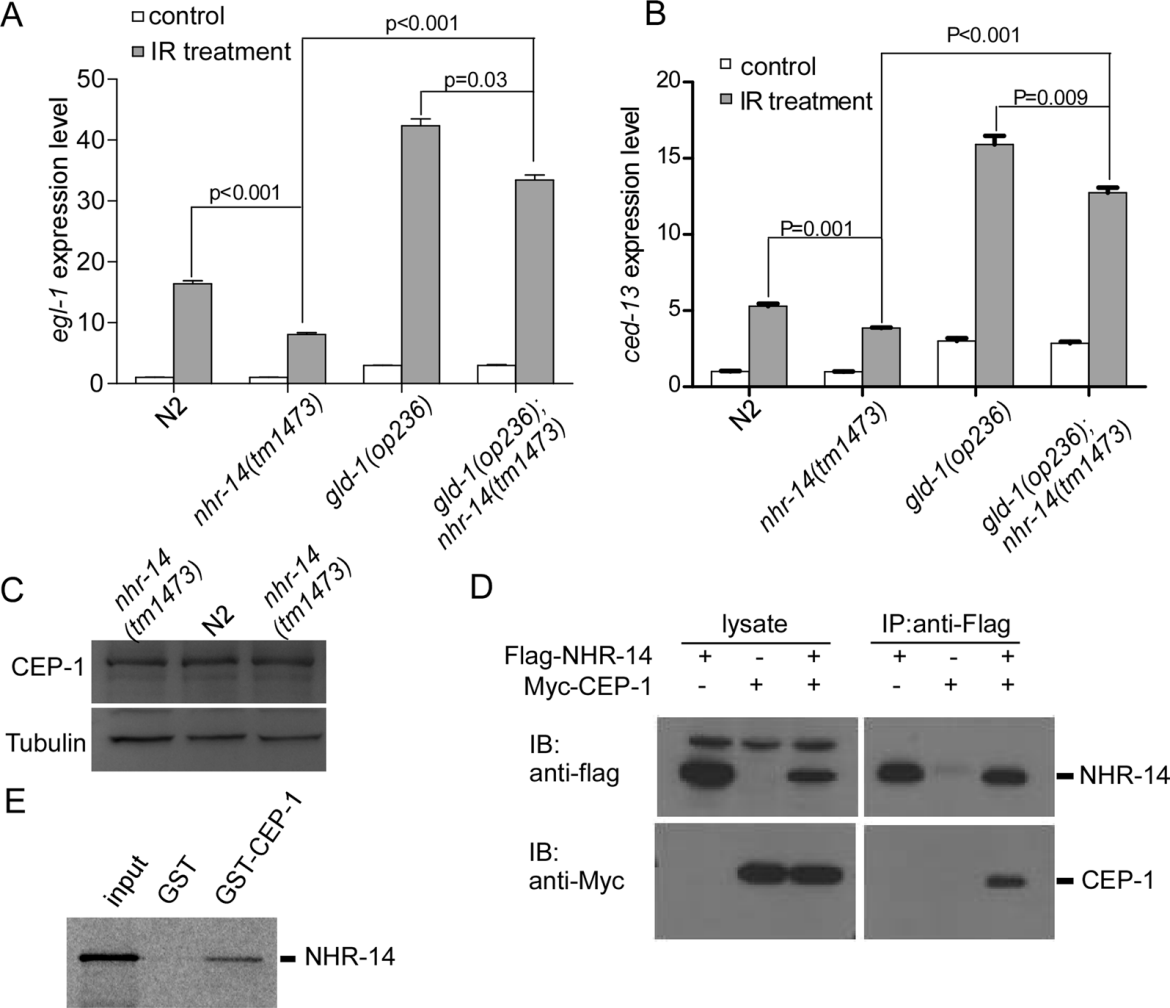
NHR-14 cooperates with CEP-1/p53 to regulate *egl-1* and *ced-13* expression. **A** Relative fold induction of *egl-1* mRNA in N2, *nhr-14(tm1473)*, *gld-1(op236)* and *gld-1(op236)*; *nhr-14(tm1473)* after 24 h of gamma irradiation (120 Gy). *egl-1* induction was averaged from three independent RT-qPCR analyses. **B** Relative fold induction of *ced-13* mRNA in N2, *nhr-14(tm1473)*, *gld-1(op236)* and *gld-1(op236)*; *nhr-14(tm1473)* following 24 h of gamma irradiation (120 Gy). *egl-onefold* change was averaged from three independent RT-qPCR analyses. **C** Western blotting analysis of CEP-1 levels in N2 and *nhr-14(tm1473)* animals. **D** NHR-14 interacts with CEP-1/p53 in mammalian cells. Flag-NHR-14 and Myc-CEP-1/p53 were co-expressed in HEK293 cells and then immunoprecipitated (IP) using Flag antibodies. The immunoprecipitated proteins were detected by immuno-blotting (IB) with Myc antibodies. E. NHR-14 and CEP-1/p53 directly interact in vitro. The full-length NHR-14 protein was in vitro translated and labeled with [.^35^S] methionine and incubated with GST or GST-CEP-1/p53 fusion proteins, which were immobilized on glutathione sepharose beads, for 2 h. After extensive washes, the bound proteins were resolved by SDS-PAGE and detected by autoradiography

To investigate how *nhr-14*/HNF4α regulates DNA damage-induced programmed cell death, we first examined the expression level of apoptotic initiator gene *egl-1* and *ced-13* in N2 and *nhr-14(tm1473)* worms. We irradiated N2 and *nhr-14(tm1473)* young adult worms at a dose of 120 Gy and performed RT-qPCR experiment, our results show that gamma-irradiation-induced *egl-1* and *ced-13* levels were significantly reduced in *nhr-14(tm1473)*. In N2 worms, the *egl-1* level was increased by 20 folds. However, in *nhr-14(tm1473)*, *egl-1* expression only increased 8 folds after DNA damage (Fig. 5A). *ced-13* expression level was induced more than fivefold in N2 worms upon gamma-irradiation, but only about threefold in *nhr-14(tm1473)* worms (Fig. 5B). These results suggest that *nhr-14*/HNF4α regulates DNA damage-induced *egl-1* and *ced-13*. To examine if *nhr-14(tm1473)* affects CEP-1 level, we performed western blotting to test CEP-1 levels in N2 and *nhr-14(tm1473)*, we found that *nhr-14(tm1473)* did not affect the protein level of CEP-1 (Fig. 5C).

Previous studies demonstrated that CEP-1/p53 is a key transcription factor of *egl-1* and *ced-13* [23, 24]. Because *nhr-14*/HNF4α regulates *egl-1* and *ced-13* at mRNA levels, we hypothesized that NHR-14/HNF4α could be a cofactor of CEP-1/p53. To this end, we first examined if NHR-14/HNF4α forms a complex with CEP-1/p53. Flag-tagged NHR-14 and Myc-CEP-1 were co-transfected into 293 T cells, and then CoIP was performed. Figure 5D shows that Myc-CEP-1/p53 was co-immunoprecipitated with Flag-NHR-14, suggesting that these two proteins interact with each other in mammalian cells. To investigate if NHR-14/HNF4α directly binds to CEP-1/p53, we performed in vitro GST-pull down assays. We found that GST-CEP-1 fusion proteins pulled-down ^[35S]^methionine labeled NHR-14 but not GST alone (Fig. 5E). We next investigated whether *nhr-14*/HNF4α regulates DNA damage-induced programmed cell death through *cep-1*/p53. As GLD-1 is a translational repressor of *cep-1*/p53 via directly binding to the 3′UTR of *cep-1*/p53 mRNA [25], *gld-1(op236)* loss-of-function mutants expresses higher levels of CEP-1/p53 in *C. elegans*. We found that *egl-1* and *ced-13* mRNA levels were much higher in *gld-1(op236)*; *nhr-14(tm1473)* double mutants than *nhr-14(tm1473)* worms after gamma-irradiation (Fig. 5A, B), which indicated that up-regulated CEP-1/p53 in *gld-1(op236)* could rescue DNA damage-induced *egl-1* and *ced-13* expression in *nhr-14(tm1473)*.

**Fig. 6:**
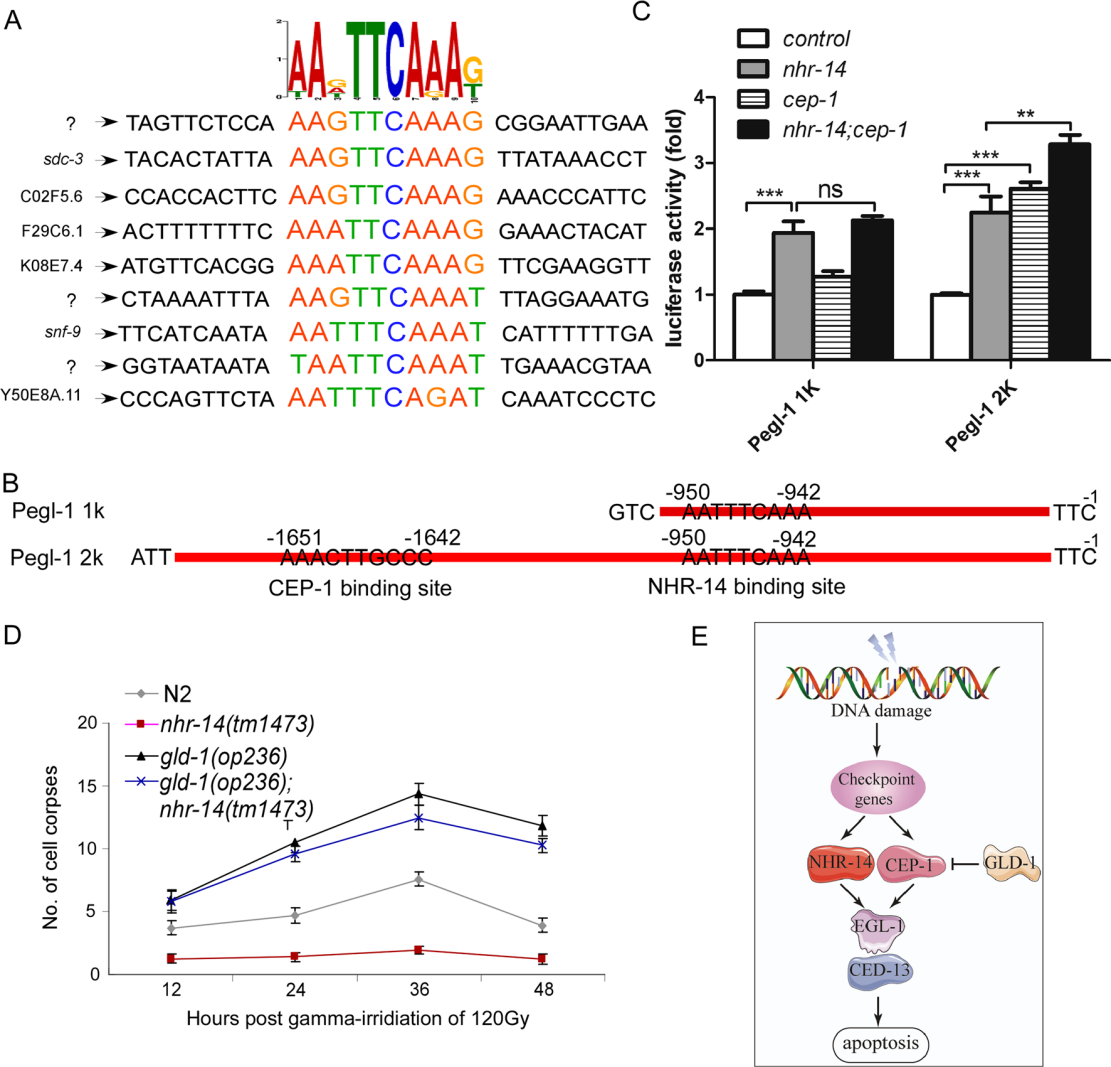
NHR-14 cooperates with CEP-1/p53 bind directly to the *egl-1* promoter and regulate DNA damage-induced apoptosis. **A** Diagram of conserved NHR-14-binding DNA sequences. Most of the DNA fragment sequences bound to NHR-14 were analyzed by meme software. **B** Binding sites of NHR-14 and CEP-1 in the *egl-1* promoter region. **C** Luciferase assay analysis of NHR-14 and CEP-1 directly bound to the *egl-1* promoter region. **D** Quantitative analysis of germ cell apoptosis induced by gamma-irradiation at indicated time points post L4 in N2, *nhr-14(tm1473)*, *gld-1(op236)* and *gld-1(op236)*; *nhr-14(tm1473).* Up-regulated CEP-1/p53 via loss function of *gld-1(op236)* rescued DNA damage-induced programmed cell death in *nhr-14(tm1473)* animals. **E** The genetic pathway for *nhr-14*/HNF4 to regulate DNA damage-induced apoptosis. NHR-14/HNF4 functions as a cofactor of CEP-1/p53 to regulate DNA damage-induced apoptosis via controlling *egl-1* and *ced-13* expression

To further confirm our result, we employed dual luciferase assays to see if coexpression of NHR-14 and CEP-1 can promote *egl-1* promoter-driven luciferase activity. We first performed the SELEX (systematic evolution of ligands by exponential enrichment) assay [13] to explore the NHR-14 bound DNA conserved sequence. After sequencing the NHR-14 binding sequence, we found that NHR-14 could bind to the” AANTTCAAA” motif (Fig. 6A), which is located on the *egl-1* promoter region between − 950 to − 942 (Fig. 6B). CEP-1 has been reported to bind to the RRRCWWGYYY motif [26, 27], which locates on the *egl-1* promoter between − 1651 to − 1642 (Fig. 6B). The luciferase assay indicated that overexpression of NHR-14 or CEP-1 can increase the *egl-1* promoter-driven luciferase activity, and coexpression of NHR-14 and CEP-1 has much higher luciferase activity than expression of NHR-14 or CEP-1 alone (Fig. 6C). These data suggest that NHR-14/HNF4α and CEP-1/p53 might directly interact with each other to regulate *egl-1* and *ced-13* transcription. However, considering that the CEP-1 and NHR-14 bindings sites are relatively far from each other, we cannot exclude the possibility that CEP-1 and NHR-14 drive *egl-1* transcription in a manner independent of their direct interaction.

We also demonstrated that up-regulated CEP-1/p53 in *gld-1(op236)* could rescue DNA damage-induced apoptosis (Fig. 6D).

In conclusion, our data suggest that *nhr-14*/HNF4α may function together with *cep-1*/p53 and regulates DNA damage-induced programmed cell death through CEP-1/p53 (Fig. 6E).

## Discussion

DNA damage-induced programmed cell death is associated with various human malignancies and identification of regulators in the DNA damage-induced apoptosis pathway is critical for intervention of these diseases. *C. elegans* has been shown to be an excellent model to study DNA damage-induced programmed cell death. And thus it is very helpful for us to understand the mechanism of carcinogenesis by studying the regulation of DNA damage-induced apoptosis in *C. elegans* germlines.

P53 is a key tumor suppressor and its mutations were detected in more than 50% of human cancers. In *C. elegans*, the p53 homolog CEP-1 acts as a key effector to mediate germ cell apoptosis triggered by ionizing irradiation [28]. Identification of new co-factors of CEP-1/p53 in *C. elegans* may offer critical targets for cancer intervention.

In response to DNA damage stimuli, the checkpoint genes will sense the signals and induce cell cycle arrest or programmed cell death. Simultaneously, CEP-1/p53 is activated and subsequently induces up-regulation of BH3 genes *egl-1* and *ced-13*. Mutation of the checkpoint genes block the transfer of DNA damage signals and reduce DNA damage-induced apoptosis. Nuclear hormone receptor family is a key to many important cellular processes, but the role of the NHR family in DNA damage-induced programmed cell death remains elusive. Previous studies showed that NHR-14/HNF4α, which was thought to be an estrogenic hormone receptor [10], was involved in the immune response processes via regulation of vitellogenin expression [29]. In the present report, we identified *nhr-*14/HNF4α as an important member of NHR in the regulation of DNA damage-induced apoptosis. Moreover, our results indicated that *nhr-*14/HNF4α is involved in regulation of the DNA damage-induced apoptosis, but not the physiological programmed cell death (Fig. 3).

Mechanically, our experiments revealed that *nhr-14*/HNF4α regulates DNA damage-induced transcription of *egl-1* and *ced-13.* More significantly, we showed that NHR-14/HNF4α interacts with CEP-1/p53 and might function as a cofactor of CEP-1/p53. However, considering that the CEP-1 and NHR-14 bindings sites are relatively far from each other, it is possible that there are shared or closely spaced CEP-1 and NHR-14 sites in the *egl-1* promoter region that we have not identified by the SELEX method. Another possibility is that CEP-1 and NHR-14 regulate *egl-1* transcription independent of their direct interaction. In addition, the *nhr-14(tm1473)* mutant dramatically reduces CEP-1/p53-mediated DNA damage-induced apoptosis. Thus we consider that *nhr-14* is a general positive regulator of DNA damage-induced germline apoptosis. Our study first reported a nuclear hormone receptor NHR-14/HNF4α that is involved in DNA damage-induced apoptosis. We have identified that NHR-14/HNF4α might cooperate with CEP-1/p53 to control DNA damage-induced *egl-1* and *ced-13* and it could provide new targets for cancer intervention.

Dysregulation of DNA damage-induced apoptosis usually leads to tumorigenesis. Nuclear receptor HNF4 alpha is one of the central elements in the liver. It was closely related to fatty acid metabolism [30–33] and can induce hepatoma differentiation and block hepatocarcinogenesis [34]. Therefore, deregulation of hepatocyte nuclear factor 4 (HNF4) could be a marker of liver cancer progression. In the future, we will further confirm the relationship between the dysregulation of DNA damage-induced apoptosis by *nhr-14*/HNF4α deletion and tumorigenesis and will further study the mechanism of HNF4α in tumorigenesis.

## Conclusions

Our study revealed a potential function of NHR-14 in DNA damage-induced apoptosis. And *nhr-14*/HNF4α functions together with *cep-1*/p53 to regulate DNA damage-induced programmed cell death.

## Data Availability

Not applicable.

## Abbreviations

NHR: Nuclear hormone receptors
HNF4: Hepatocyte nuclear factor
PRMT: Protein arginine methyltransferase
UTR: Untranslated regions
PMSF: Phenylmethylsulfonyl fluoride
SDS-PAGE: Sodium dodecyl sulfate polyacrylamide gel electrophoresis
SELEX: Systematic evolution of ligands by exponential enrichment

## References

[1] Antebi A (2006). Nuclear hormone receptors in *C. elegans*. WormBook.

[2] Magner DB (2013). The NHR-8 nuclear receptor regulates cholesterol and bile acid homeostasis in *C. elegans*. Cell Metab.

[3] Verghese E (2011). The tailless ortholog nhr-67 functions in the development of the *C. elegans* ventral uterus. Dev Biol.

[4] Goudeau J (2011). Fatty acid desaturation links germ cell loss to longevity through NHR-80/HNF4 in *C. elegans*. PLoS Biol.

[5] Liang B (2010). The role of nuclear receptor NHR-64 in fat storage regulation in *Caenorhabditis elegans*. PLoS ONE.

[6] Gissendanner CR (2008). The *Caenorhabditis elegans* NR4A nuclear receptor is required for spermatheca morphogenesis. Dev Biol.

[7] Gissendanner CR (2014). *C. elegans* nuclear receptor NHR-6 functionally interacts with the jun-1 transcription factor during spermatheca development. Genesis.

[8] Peterson ND (2019). The nuclear hormone receptor NHR-86 controls anti-pathogen responses in *C. elegans*. PLoS Genet.

[9] Van Gilst MR (2005). Nuclear hormone receptor NHR-49 controls fat consumption and fatty acid composition in *C. elegans*. PLoS Biol.

[10] Mimoto A (2007). Identification of an estrogenic hormone receptor in *Caenorhabditis elegans*. Biochem Biophys Res Commun.

[11] Yang M (2009). Caenorhabditis elegans protein arginine methyltransferase PRMT-5 negatively regulates DNA damage-induced apoptosis. PLoS Genet.

[12] Schumacher, B., et al., *The C. elegans homolog of the p53 tumor suppressor is required for DNA damage-induced apoptosis.* Curr Biol, 2001. **11**(21): p. 1722–7. 10.1016/s0960-9822(01)00534-6 11696333

[13] Jian Y (2009). RNA aptamers interfering with nucleophosmin oligomerization induce apoptosis of cancer cells. Oncogene.

[14] Quevedo C, Kaplan DR, Derry WB (2007). AKT-1 regulates DNA-damage-induced germline apoptosis in *C. elegans*. Curr Biol.

[15] Hengartner MO, Horvitz HR (1994). *C. elegans* cell survival gene ced-9 encodes a functional homolog of the mammalian proto-oncogene bcl-2. Cell..

[16] Seshagiri S, Miller LK (1997). Caenorhabditis elegans CED-4 stimulates CED-3 processing and CED-3-induced apoptosis. Curr Biol.

[17] Spector MS (1997). Interaction between the *C. elegans* cell-death regulators CED-9 and CED-4. Nature.

[18] Adamo A (2008). BRC-1 acts in the inter-sister pathway of meiotic double-strand break repair. EMBO Rep.

[19] Boulton SJ (2004). *BRCA1*/*BARD1 orthologs required for DNA repair in Caenorhabditis elegans*. Curr Biol.

[20] Zhou Z, Hartwieg E, Horvitz HR (2001). CED-1 is a transmembrane receptor that mediates cell corpse engulfment in *C. elegans*. Cell.

[21] Xiao H (2009). Lysosome biogenesis mediated by vps-18 affects apoptotic cell degradation in *Caenorhabditis elegans*. Mol Biol Cell.

[22] Gartner A (2000). A conserved checkpoint pathway mediates DNA damage–induced apoptosis and cell cycle arrest in *C. elegans*. Mol Cell.

[23] Hofmann ER (2002). *Caenorhabditis elegans* HUS-1 is a DNA damage checkpoint protein required for genome stability and EGL-1-mediated apoptosis. Curr Biol.

[24] Schumacher B (2005). *C. elegans* ced-13 can promote apoptosis and is induced in response to DNA damage. Cell Death Differ.

[25] Schumacher B (2005). Translational repression of *C. elegans* p53 by GLD-1 regulates DNA damage-induced apoptosis. Cell.

[26] Xu D (2014). Analysis of the p53/CEP-1 regulated non-coding transcriptome in *C. elegans* by an NSR-seq strategy. Protein Cell.

[27] Huyen Y (2004). Structural differences in the DNA binding domains of human p53 and its *C. elegans* ortholog Cep-1. Structure.

[28] Han Z (2005). The *C. elegans* Tousled-like kinase contributes to chromosome segregation as a substrate and regulator of the Aurora B kinase. Curr Biol.

[29] Fischer M (2012). Phytoestrogens genistein and daidzein affect immunity in the nematode *Caenorhabditis elegans* via alterations of vitellogenin expression. Mol Nutr Food Res.

[30] Duda K, Chi YI, Shoelson SE (2004). Structural basis for HNF-4alpha activation by ligand and coactivator binding. J Biol Chem.

[31] Dhe-Paganon S (2002). Crystal structure of the HNF4 alpha ligand binding domain in complex with endogenous fatty acid ligand. J Biol Chem.

[32] Hertz R (1998). Fatty acyl-CoA thioesters are ligands of hepatic nuclear factor-4alpha. Nature.

[33] Wisely GB (2002). Hepatocyte nuclear factor 4 is a transcription factor that constitutively binds fatty acids. Structure.

[34] Wei L (2017). Oroxylin A activates PKM1/HNF4 alpha to induce hepatoma differentiation and block cancer progression. Cell Death Dis.

